# Pure Polyphenols and Cranberry Juice High in Anthocyanins Increase Antioxidant Capacity in Animal Organs

**DOI:** 10.3390/foods8080340

**Published:** 2019-08-12

**Authors:** Tracy Bariexca, Janice Ezdebski, Benjamin W. Redan, Joe Vinson

**Affiliations:** 1 Department of Chemistry, Loyola Science Center, University of Scranton, Scranton, PA 18510, USA; 2 Center for Food Safety and Applied Nutrition, Office of Food Safety, Division of Food Processing Science and Technology, U.S. Food and Drug Administration, 6502 South Archer Road, Bedford Park, IL 60501, USA

**Keywords:** polyphenols, anthocyanins, cranberry juice, antioxidant capacity, organs

## Abstract

Anthocyanins and the broader class of polyphenols are strong antioxidants in vitro. Polyphenols are one of the major antioxidants in plant foods, and the beverages derived from them. There is extensive evidence in the literature that polyphenols are beneficial to health. In order to be bioactive in vivo, they need to be bioavailable and be transported from the circulation to target organs. To date, there have been few studies testing the extent to which polyphenols and especially anthocyanins affect the antioxidant capacity of animal organs. In our first pilot study, we investigated how three pure polyphenols (the flavonoids quercetin, catechin and hesperetin) given to rats by intraperitoneal injection (49 to 63 mg/kg) affected their organ antioxidant capacity. This was followed by a subsequent study that injected one ml of 100% cranberry juice (high in anthocyanins) to hamsters. Antioxidant capacity of animal organs was determined by using the ferric reducing antioxidant power (FRAP) colorimetric assay on methanolic extracts of select rat organs (i.e., liver, kidney, heart, prostate and brain) and in the hamster organs (i.e., liver, kidney, heart, bladder and brain). Overall the results showed that antioxidant capacity was significantly increased (*p* < 0.05) in experimental vs. control organs. Analysis of organs by high performance liquid chromatography (HPLC) from both animal studies provided evidence of polyphenol metabolites in the organ extracts. Taken together, this study provides data that the administration of anthocyanins and other polyphenols cause an increase in organ antioxidant capacity in two animal models. This result supports the growing evidence for the hypothesis that dietary polyphenols reduce the risk and extent of various chronic disease at the disease site.

## 1. Introduction

Polyphenols (PP) are a large group (10,000 and growing) of plant secondary metabolite, compounds that can be broadly divided into flavonoids and non-flavonoids [1]. The non-flavonoids include such compounds as the phenolic acids (major phenolics in coffee and grains) and others such as the heavily studied minor compound in red wine, resveratrol. Flavonoids include the flavonol quercetin (fruits, vegetables), the flavanol catechin (tea) and the flavanone hesperetin (citrus fruit). Red wine and other fruit juices such as cranberry juice (CJ) are high in the flavonoids known as anthocyanins. Our group has shown that cranberry has the highest fresh weight concentration of polyphenols among the commonly consumed fruits in the USA [2].

As determined by our antioxidant capacity assay commercial 100% CJ ranks the highest among commonly consumed fruit juices [3]. With the exception of high vitamin C fruits such as oranges, PP are the major antioxidants in plant foods with the green tea leaf containing concentrations as high as 25 weight % [4]. Although global PP intake varies widely, the overall dietary contribution from PP is estimated to be ~1 g/day [5]. Dietary intake of anthocyanins has been estimated to be 11 mg/day from the USDA Database on Flavonoids and two other publicly available databases on food consumption in the USA [6].

Data on the health aspects of PP and anthocyanins are primarily from epidemiological studies. The PREDIMED study showed an interventional diet rich in PP [7,8] resulted in significantly lowering of the risks of cardiovascular disease (CVD), diabetes, and the incidence of overall mortality. In the US Nurses’ Health Study there was an inverse association of the consumption of flavonoid-rich foods and specific flavonoid subclasses with all-cause mortality [9]. Further epidemiological data illustrate an association between anthocyanin intake and reduced risk of myocardial infarction and CVD related mortality [10].

In light of these data linking PP intake to disease residence, it is important to conduct controlled studies. The purpose of this investigation is to show that both pure PP and the anthocyanins in CJ are absorbed after intraperitoneal injection (i.p.) and can enhance the antioxidant capacity in animal organs where chronic disease such as heart disease, diabetes, and neurological aging diseases such as Alzheimer’s are manifested. This i.p. method of dosing was used to ensure maximum PP bioavailability. Two rodent models were used in this work; first, rats because they are the most studied animal model for PP investigations, and second, hamsters were used since they have been extensively used by our laboratory and others to investigate the impact of PP consumption in the form of extracts, foods and beverages on atherosclerosis.

## 2. Materials and Methods

### 2.1. PP and CJ

The first study examined pure PP (Sigma-Aldrich, St. Louis, MO USA) of three different subclasses. PP solutions or suspensions were made by vortexing in water. Concentrations were 19.4 mg/mL quercetin (flavonol), 15.0 mg/mL (+)-catechin (flavanol), and 15.0 mg/mL hesperetin (flavanone). Fresh solutions/suspensions were made each day for the rat study and used on the same day as prepared. PP metabolites 3′-O-methylcatechin and quercetin-3-sulfate were synthesized and purified in-house. The second study used Knudsen’s (Just Cranberry) 100% CJ available from a local health food store. This brand of CJ is more concentrated than the 27% CJ cocktail found at most grocery stores. Total PP were measured by a standard colorimetric method which uses the single reagent Folin assay at 750 nm after acid hydrolysis of bound PP [11,12] and catechin as the standard. The anthocyanin concentration in the CJ was calculated by the standard pH differential method [13].

### 2.2. Animal Studies (Protocols #3-06 and #8-0 Were Approved by the University of Scranton Animal Committee)

Adult Sprague-Dawley rats (Charles River Laboratories. Wilmington, MA USA) weighing 308 ± 81 g were acclimated in single cages and fed *ad libitum* with Purina Rodent Chow for one week. The room temperature and humidity were controlled and held constant (16 °C and 22%, respectively). A 12-h light cycle was maintained for one week before PP dosing. There were three experimental groups of 7 rats and a control group of 7 rats. A one ml suspension of either quercetin, catechin, hesperetin, or water (control) was administered by intraperitoneal (i.p.) injection with a syringe each day for three days. On day 3, the rats were sacrificed 2 h after injection. Sacrifice was performed by giving sodium pentobarbital solution. The animal was fixed on a dissecting board, the body cavity was opened, and the right atrium clipped followed by perfusion of the systemic circulation with a saline solution injected through the descending aorta until the liver appeared to be free from blood. The organs were quickly perfused with saline to remove blood. Harvest organs were snap frozen in liquid nitrogen and stored at −80 °C until the assay. The organs were weighed and a section of liver, kidney and brain (~1 g), ~0.2 g of prostate and ~0.5 g of heart was removed by surgical scissors and weighed. The larger rat organs were homogenized with a mortar and pestle in 10 mL of HPLC-grade methanol at 4 °C while 2 mL was used for the prostate and 5 mL for the heart. Rat organ homogenates were placed in a 10 mL plastic centrifuge tube to which 20–100 µL of pure acetic acid was added. Acidified organ extracts were flushed with nitrogen, centrifuged at 4 °C and supernatant was collected and stored at −80 °C.

In the hamster study 14 Syrian golden hamsters (Charles River Laboratories, average weight (157 ± 11 g) were fed powdered rodent chow ad libitum and acclimated to the animal room for one week. One ml of CJ or one ml of water (control) was administered i.p. to two groups of 7 hamsters daily for 7 days. On day 7 they were given the treatments and anesthetized 2 h after the i.p. injections. After blood was removed from the heart by a needle rinsed with EDTA solution, organs were removed and perfused with saline. Urine was removed from the bladder with a syringe and the bladder rinsed with saline. The organs, were flash frozen with liquid nitrogen and stored at −80 °C. Hamster organs were homogenized at 4 °C with methanol: HCl (99:1; *v*:*v*) flushed with nitrogen, centrifuged, and then frozen as with the rat organs. The organ weights in the controls and in experimental groups were not significantly different (*p* > 0.05) for both the rat and hamster study.

#### 2.2.1. Organ Antioxidant Capacity (AC) Analysis

(+)-Catechin was used as the standard for AC measurements of the organ extracts. A stock standard was prepared in methanol and diluted to 10–100 µM in the cuvette for the standard curve. Methanol was used as the blank. Ferric Reducing Antioxidant Power (FRAP) assay was used for AC analysis following the classic Benzie paper [14]. A Genesys 20 spectrophotometer (Thermo Scientific) was used for all colorimetric absorbance readings at 593 nm.

#### 2.2.2. HPLC Analysis of Organ Extracts

UV/Vis High Performance Liquid Chromatography (HPLC) was used to separate and identify extracted PP and their metabolites. A Shimadzu Prominence HPLC system (LC-20AD) equipped with a Diode Array detector (SPD-M20A) was used for the rat organs analysis and a single wavelength detector (SPD-10AV) at 280 nm for the hamster organ analysis. The hamster CJ study was conducted at a time when the diode array detector was unavailable. A much later examination of the hamster organ extracts revealed degradation of the PP and no further analysis with the diode array detector was possible. A Phenomenex Hypersil C18 column (4.5 × 250 mm; 10 µm particle size) was used for the separations. The gradient separations were developed in-house. For rat PP metabolites, Solvent A was HPLC grade water with 2% acetic acid and Solvent B was methanol with 2% acetic acid. The gradient was initially 10% B, 50% B after 15 min and 100% B at 25 min with a flow rate was 0.4 mL/min The PP were detected at 280 nm. For hamster CJ metabolites the mobile phases were Solvent A, HPLC grade water/1% H3PO4 and Solvent B, HPLC grade methanol/1% H3PO4.This acidic solvent ensured stability of anthocyanins and gave a better separation. The flow rate was 0.5 mL/min. The gradient system was 0% B at 0 min, 50% B at 15 min, and 100% B at 30 min with the detector set at 520 nm.

### 2.3. Data Analysis

All FRAP analysis was done in duplicate for each of the samples of organ assayed and the average and standard deviation were determined for each group. Sigma Stat 3.0 (Systat, San Jose, CA USA) was used for the statistical analysis. Statistical significance were set at *p* < 0.05. After ANOVA followed by a Tukey’s post-hoc test for significant difference within groups, the student’s t-test was used to determine if two normally distributed groups were significantly different from each other or the Mann-Whitney test for two non-normally distributed groups.

## 3. Results

### 3.1. Rat Study with Pure PP Compounds

#### 3.1.1. Organ AC

The PP dose given to the rats is in the range of most comparable studies with a similar animal model (10–100 mg/kg). The multiple dosing over a period of days allowed organ accumulation of the PP and potential antioxidant changes. Table 1 shows the changes in FRAP AC in 5 different organs after the rats received the PP treatment.

The control brain (18.4 µM) had the lowest antioxidant capacity of the organs by a factor of 2 to greater than 10 depending on the PP type. The difference in AC between brain and the other organs was highly significant (*p* < 0.001) and was probably a result of the blood-brain barrier limiting PP accumulation. In another study, the brain also had the lowest AC among the organs in a rats given a green tea extract high in catechins and other PP [15]. Another analysis showed that FRAP AC of brain tissue was lowest compared to the other organs tested [16]. In an animal study that fed quercetin to pigs, the brain had the lowest total quercetin metabolite concentrations among the organs analyzed by HPLC [17]. Interestingly, our work shows that the prostate had an AC value significantly higher than the other organs (*p* < 0.001). One study found that metabolites of catechins accumulate in the prostate of mice and humans after green tea consumption [18]. Hesperetin alone or as naturally present in orange juice was found to increase serum AC when given to rats [19]. In our study the rank order of organ AC was PP dependent and varied across organs. The liver showed the greatest % increase in the FRAP value for the treated animals vs. the control, with an 82% increase in the catechin group. Hesperetin treatment as compared to the other PP resulted in the largest percent increase in organ FRAP values.

#### 3.1.2. HPLC of PP Metabolites

**Figure 1:**
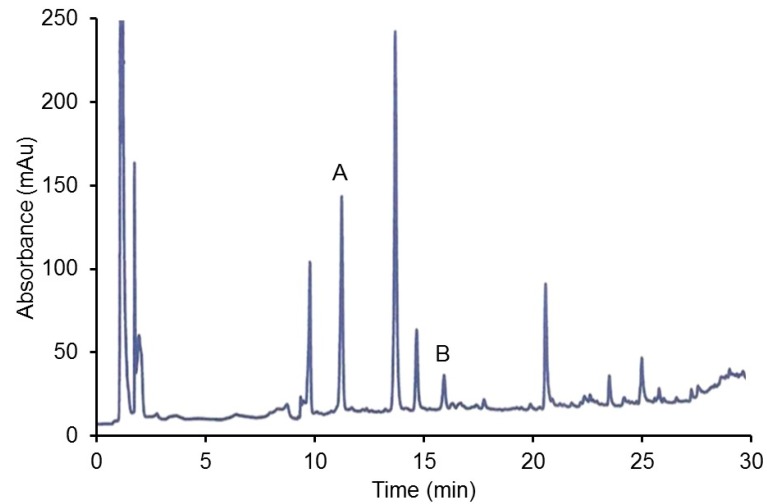
HPLC of kidney extract from rat administered quercetin (A = sulfonated quercetin, B = quercetin).

**Figure 2:**
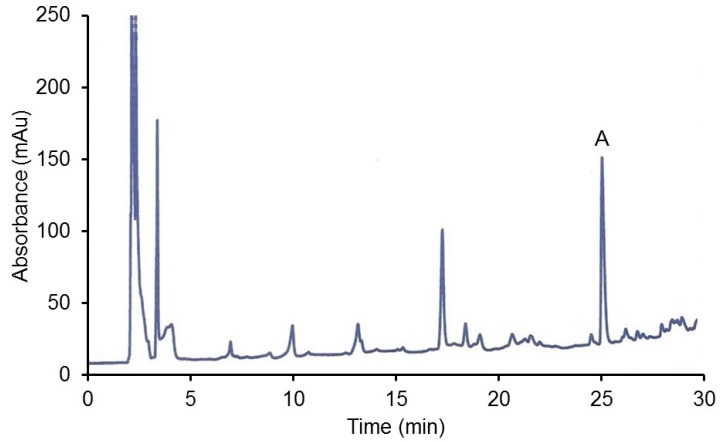
HPLC of liver extract of rat administered catechin (A = methylated catechin).

The HPLC chromatograms of two of the methanolic organ extracts are shown in Figure 1 and Figure 2. In Figure 1 it can be seen (assuming a similar extinction coefficient) that there is much more sulfonated metabolite (quercetin-3-O-sulfate) than the original aglycone quercetin administered to the rats. This is consistent with the normal pattern of PP metabolism in both animals and humans. The peaks with a retention time prior to 2 min were hypothesized to be phenolic acids in the grains present in the animal food.

Figure 2 shows that the major PP metabolite of catechin is the methyl catechin, matching the 3′-O-methylcatechin standard’s retention time and spectrum. This metabolite in the glucuronidated form was also found in the liver, kidney, brain and heart of rats given a very high dose of catechin (500 mg/kg body weight) with the collection 2 h after administration [18]. The i.p. injection, as compared to gavage, maximizes the amount of PP absorbed since it bypasses the digestion system which can results in considerable PP degradation. The peaks appearing at lower retention time (after 3 min and before 12 min) in the figure are presumably the more polar water soluble glucuronidated/sulfonated methyl catechins as reported [18]. No unmetabolized catechin was observed, which is consistent with previous catechin studies [18].

In the above figure, the largest peak is the 3-O-methylcatechin and presumably glucuronidated/sulfated catechin metabolites are at the earlier retention times (after 3 min and before 26 min).

### 3.2. CJ Study

#### 3.2.1. CJ Analysis

FRAP analysis after acid hydrolysis indicated that the CJ contained 6.8 mM total PP (as catechin equivalents) and the pH differential method found 2.1 mM anthocyanins. Thus CJ had approximately 31% of its PP as anthocyanins. HPLC analysis after acid hydrolysis showed the anthocyanins cyanidin and peonidin, which is consistent with other literature [20]. The 1 mL dose of CJ provided 931 μg anthocyanidins (cyanidin-3-glycoside equivalents) to the hamsters. The dose to the hamsters from CJ was thus 5.9 mg/kg body weight anthocyanins and 19.0 mg/kg total PP as catechin equivalents. Thus 1 mL of CJ provides a very high dose of anthocyanins compared to average US intake of 11 mg/70 kg body weight (6).

#### 3.2.2. CJ Hamster Organs AC

**Figure 3:**
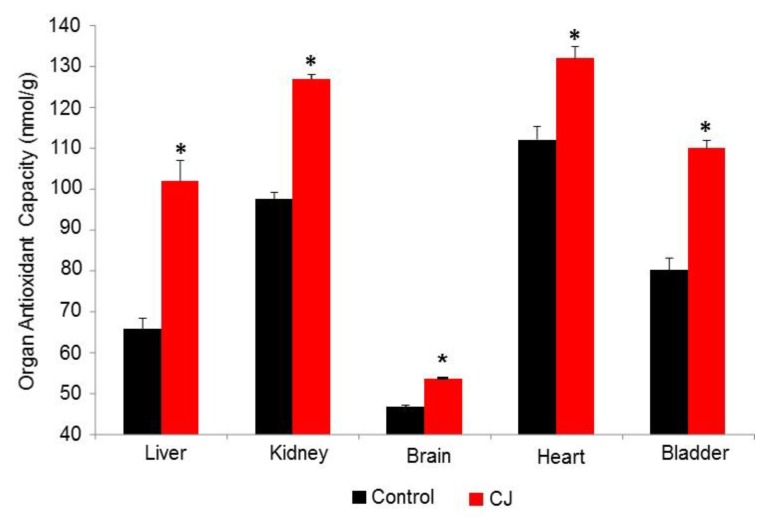
Hamster Organ Antioxidant Capacity after CJ administration. * *p* < 0. 0001 vs. the control.

The results of the organ extract FRAP analysis after administration of CJ are shown in Figure 3. As opposed to the PP organ analysis, the variations within the groups were quite small. This may be attributed to the fact that the PP were given as a suspension to the rats while CJ was a solution. The FRAP value rank order for the control group was heart > kidney > bladder > liver >> brain. As was the case in the rat study the brain FRAP value was significantly lower than the other organs for the hamster. CJ treatment caused significant increases in AC for all the organs. Among the organs tested, the liver showed the greatest % change in FRAP with CJ administration.

#### 3.2.3. HPLC Analysis of Hamster CJ Organs

The organs appeared slightly reddish presumably due to the high concentrations of anthocyanins. In the HPLC chromatograms (not shown because of poor printer quality) the anthocyanins are detected at retention times earlier than the endogenous peaks (presumably heme proteins) at 24+ min These early peaks are probably anthocyanin metabolites since the anthocyanin glycosides in CJ occurred at 24–25 min and would not be distinguishable from the endogenous peaks. The i.p. injection of CJ should eliminate the complete colonic metabolism of the anthocyanins to phenolic acids as seen in rats [21] and humans [22] after low dose oral consumption. In the aformentioned studies no anthocyanins were detected in plasma or urine. However, rats provided a very high dose of blackberry anthocyanins (3000 mg/kg) were found to have anthocyanin glycosides and their metabolites in the prostate and heart [23]. In a later study by the same investigators anthocyanin metabolites were found in the liver, kidney and brain [24]. In mice, treatment with purple tea anthocyanins increased brain AC which was attributed to anthocyanin metabolites found in the brain [25].

## 4. Discussion

It is important to investigate how administering PP as either pure compounds of a food or beverage (such as CJ) affects the AC of various organs. In chronic diseases such as heart disease and cancer there is extensive oxidative damage (lipid and protein oxidation) that occurs in the organs Since PP are strong in vivo antioxidants, these compounds can potentially counter the oxidative stress through direct chemical action or through activation of other cell signaling pathways involved in antioxidant response. In most animal organ studies involving pure PP administration, the AC of the control group was between 1-10 µmol/kg using ferrous ion as the standard for FRAP. In our study standard and the control organs’ AC for rats and hamsters were between 10–100 µmol/kg. In previous published studies, actual PP concentrations in the organs of single PP gavaged rodents ranged from 1–10 µM. Thus, our studies showed a larger control AC and larger AC increase from the treatment than might be expected based solely on the presence of PP. We hypothesize that this larger increase could be partly due to the i.p. method of administration which is comparable to i.v. injection with 100% absorption of the PP. The AC increase could also be due to up-regulation of synthesis of antioxidants present in organs such as ascorbic acid, whose values in rodent organs can be in the hundreds of µM [26]. We have also found that oral intake citrus PP with ascorbate caused a significantly greater increase in human plasma ascorbate than vitamin C given alone. We hypothesized that this synergistic effect may be due to PP sparing ascorbate by preventing oxidation in the body [27]. Lastly PP could increase antioxidant enzymes and glutathione levels in the organs and thus spare PP.

Organ AC as determined by FRAP is the sum of all the redox active antioxidants in the extract. Albumin, uric acid, and the antioxidant enzymes present in plasma and organs could be excluded as to contributing to AC, since they would be precipitated during the methanol extraction process. Glutathione is also unreactive in the FRAP assay [14]. This is the first study to determine the effect of CJ administration on AC of animal organs. A previous study using a rat model found that drinking CJ for 4 months caused an increase in plasma AC [28]. There have been several human clinical studies that tested how CJ affected plasma AC. A single dose (240 mL of 27% CJ) increased the plasma AC temporally and countered the decrease in AC caused by the high fructose sugars given to the human subjects as a control [3]. A 2 week CJ supplementation study with 7 mL/kg bodyweight given to men, increased plasma AC and decreased oxidized LDL levels, a heart disease risk factor [29]. It is also of importance that the cranberry anthocyanin metabolites accumulate in the kidney and bladder since cranberry and especially CJ are well known to benefit urinary tract health [30]. The fact that PP metabolites also get into other organs such as the prostate [31], brain and liver are significant as growing evidence that cranberries may have a role in cancer prevention and treatment [32]. Future studies by our group include a hamster atherosclerosis investigation with CJ in the drinking water.

## 5. Conclusions

Together, these results demonstrate that administration of both pure PP and mixtures of PP in a whole-food context were both able to significantly increase the AC of both rat and hamster organs. One of the hallmarks of chronic diseases is an increased production of deleterious free radicals which are hypothesized to be neutralized by reaction with endogenous antioxidants such as glutathione, the antioxidant vitamins, as well the PP from plant foods and beverages. This data help provide support for potential mechanisms of action on how consumption of foods rich in PP are able to reduce the risk of chronic disease.

## Figures and Tables

**Table 1:** FRAP Antioxidant Capacity (µmol/kg. i.e., µM) in Different Rat Organs after Three Separate PP were Administered to Three Different Groups.

Polyphenols	Liver	Kidney	Prostate	Brain	Heart
Control	49.3 ± 3.0	75.2 ± 8.0	224 ± 77	18.4 ± 2.8	70.0 ± 11.1
Quercetin	80.1 ± 8.8 **	124 ± 9 ***	255 ± 50	13.9 ± 3.9	82.3 ± 12.1
Hesperetin	82.4 ± 6.0 **	148 ± 55 **	282 ± 8	19.5 ± 5.5	80.8 ± 12.4
Catechin	89.2 ± 5.3 **	131 ± 6 ***	193 ± 24	27.5 ± 8.0 *	73.0 ± 6.5

* *p* < 0.05 vs. control; ** *p* < 0.01 vs. control; *** *p* < 0.001 vs. control.
